# Influencing factors of noninvasive positive pressure ventilation in the treatment of respiratory failure: a 10-year study in one single center

**DOI:** 10.1186/s40001-021-00615-6

**Published:** 2021-12-03

**Authors:** Juan Wang, Shuang Bian, Xiaomiao Tang, Sheng Ye, Shen Meng, Wei Lei

**Affiliations:** 1 Department of Pulmonary and Critical Care Medicine, The First Affiliated Hospital of Soochow University, Suzhou, Jiangsu China; 2 Department of Internal Medicine, Weiting Community Health Service Center, Suzhou, Jiangsu China

**Keywords:** Noninvasive positive pressure ventilation, Respiratory failure, Influencing factors

## Abstract

**Background:**

The utilization of noninvasive positive pressure ventilation (NPPV) is becoming more and more common, especially in patients with acute or chronic respiratory failure. The purpose of our study is to analyze the factors that influence the efficacy of NPPV in the treatment of respiratory failure caused by a variety of etiology.

**Methods:**

From May 2011 to April 2020, patients treated with NPPV during hospitalization in the First Affiliated Hospital of Soochow University were enrolled. According to the clinical outcome of NPPV treatment and whether converted to invasive mechanical ventilation, patients were divided into the success group and the failure group. The clinical data and the characteristics of NPPV application were compared between the two groups.

**Results:**

A total of 3312 patients were enrolled, including 2025 patients in the success group and 1287 patients in the failure group. Univariate analysis suggested that there were no statistical differences in patients' age, gender, use of analgesia and/or sedation, complicated with barotrauma, inspiratory positive airway pressure and expiratory positive airway pressure between the success and failure groups (*P* > 0.05). However, there were statistically significant differences in serum albumin levels, Ca^2+^ concentration, blood glucose levels, duration of NPPV treatment and length of hospital stay between the success and failure groups (*P* < 0.05). Multivariate logistic regression analysis indicated that serum albumin levels and duration of NPPV treatment had statistical significance on the therapeutic effect of NPPV (*P* < 0.05).

**Conclusion:**

Serum albumin levels and duration of NPPV treatment were independent risk factors for the efficacy of NPPV treatment in respiratory failure.

## Background

With advances in technology and experience, NPPV has been widely used in patients with acute or chronic respiratory failure caused by various reasons, including acute exacerbation of chronic obstructive pulmonary disease (AECOPD), stable COPD, acute cardiogenic pulmonary edema (ACPE), weaning from invasive mechanical ventilation (IMV), acute respiratory distress syndrome (ARDS), obesity hypoventilation syndrome (OHS), etc. An international survey reported that the utilization rate of NPPV in general wards was 66%–73%, and that in intensive care unit (ICU) was over 90% [1]. NPPV was chosen as initial ventilatory support in approximately 40% of patients with acute respiratory failure and up to 80% of patients with AECOPD or ACPE [2].

Various factors have been reported to influence the efficacy of NPPV in the treatment of respiratory failure. Yalcinsoy et al. [3] believed that professional team and the determination of the failure criteria of NPPV were the key for the success of treatment. Some previously published studies pointed out that many factors could affect the efficacy of NPPV treatment, such as the underlying disease, age, body mass index, heart rate, respiratory rate, pH, arterial partial pressure of carbon dioxide (PaCO_2_), partial arterial oxygen pressure to the inspired fractionated oxygen (PaO_2_/FiO_2_) ratio, albumin, Ca^2+^, blood glucose, acute physiology and chronic health evaluation II (APACHE II) score < 29, glasgow coma scale (GCS) score, duration of NPPV treatment and length of hospital stay (LOS) [4–13]. Reasonable application of NPPV can overcome positive pressure ventilation airway resistance, reduce respiratory rate and the work of breathing, decrease the rate of endotracheal intubation and mortality rate, improve oxygenation and shorten LOS [14].

There are many studies on the influencing factors of NPPV in the treatment of respiratory failure caused by a single disease, especially AECOPD, ACPE, stable COPD, ARDS. However, to our best knowledge, few researches are reported on the influencing factors of the efficacy of NPPV in the treatment of respiratory failure caused by a variety of etiology. Therefore, this study aims to analyze the factors that influence the efficacy of NPPV in the treatment of respiratory failure in a single center for 10 years, with the expectation of early intervention on relevant factors in the clinical practice to improve the efficacy of NPPV treatment.

## Methods

### Patients

In this retrospective study, patients treated with NPPV during hospitalization in the First Affiliated Hospital of Soochow University from May 2011 to April 2020 were enrolled.

### Inclusive criteria

1. Patients received NPPV treatment during the above-mentioned hospitalization;
2. PaO_2_ < 60 mmHg with or without PaCO_2_ > 50 mmHg;
3. The medical records were complete and could be analyzed.

### Exclusive criteria

1. Patients with a hospitalization duration ≤ 3 days;
2. Patients under the age of 18;
3. Incomplete medical records could not be analyzed.

### Grouping criteria

According to the clinical outcome of NPPV treatment and whether converted to IMV treatment, patients were divided into the success group and the failure group:

1. Success group: (1) Patients treated with NPPV experienced remission and were discharged from hospital without converted to IMV treatment during hospitalization; (2) Patients experienced remission and were discharged after NPPV treatment as a weaning strategy for IMV. 2. Failure group: (1) Patients who did not experience remission after NPPV treatment were converted to IMV treatment; (2) Patients treated with NPPV were not converted to IMV treatment, and the clinical outcome was discharged against medical advice or death; (3) Patients were treated with NPPV as a weaning strategy for IMV, and the clinical outcome was discharged against medical advice or death.

### Research methods

The clinical data of the patients (age, gender, underlying disease, use of analgesia and/or sedation, complicated with barotrauma, serum albumin levels, Ca^2+^ concentration, blood glucose levels, LOS) and the characteristics of NPPV application (inspiratory positive airway pressure [IPAP], expiratory positive airway pressure [EPAP], duration of NPPV treatment) were recorded by retrospective study method, and the above data were analyzed statistically.

To reduce the probability that serum albumin levels, Ca^2+^ concentration and blood glucose levels were affected by the treatment, we focused on the results performed within 24 h of admission, although the above laboratory data did not vary significantly during later observation.

### Statistical analysis

SPSS 25.0 software was used for statistical analysis of data. Measurement data of non-normal distribution was represented by *M* (*P*_25_–*P*_75_), and comparison between groups was performed by Mann–Whitney *U* test. Measurement data of normal distribution or approximate normal distribution was expressed by $\bar{\chi } \pm \text{s}$, and *t* test was used for comparison between groups. Enumerative data was expressed in the form of frequency and rate, and *χ*^2^ or Fisher exact test was used for comparison between groups. Multivariate logistic regression analysis was performed on the indicators that were statistically significant in univariate analysis to explore independent risk factors for the efficacy of NPPV treatment. *P* < 0.05 was used to determine that the difference was statistical difference.

## Results

### Basic characteristics of patients treated with NPPV

**Fig. 1:**
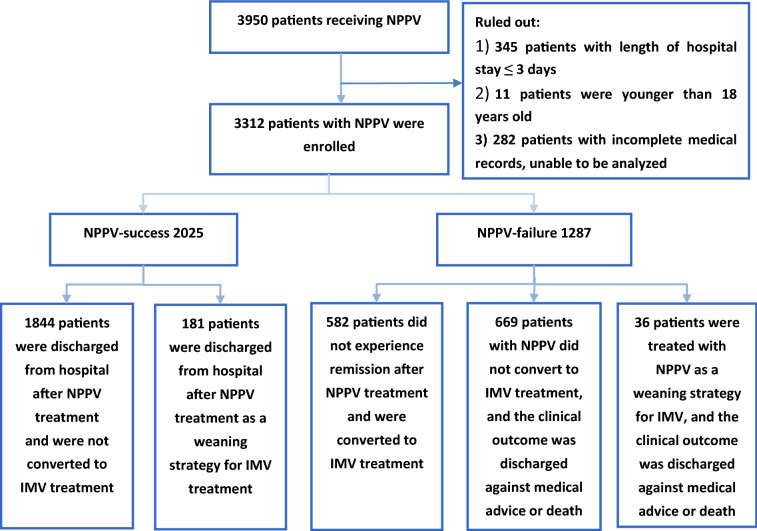
Patients flow chart. *NPPV* Noninvasive positive pressure ventilation, *IMV* Invasive mechanical ventilation

A total of 3950 patients received NPPV treatment were included, and 638 patients meeting the exclusive criteria were excluded over a 10-year period. 3312 patients received NPPV treatment were enrolled, including 2250 males and 1062 females, aged 18–104 years, with an average age of 68.00 ± 15.18 years (Fig. 1).

**Fig. 2:**
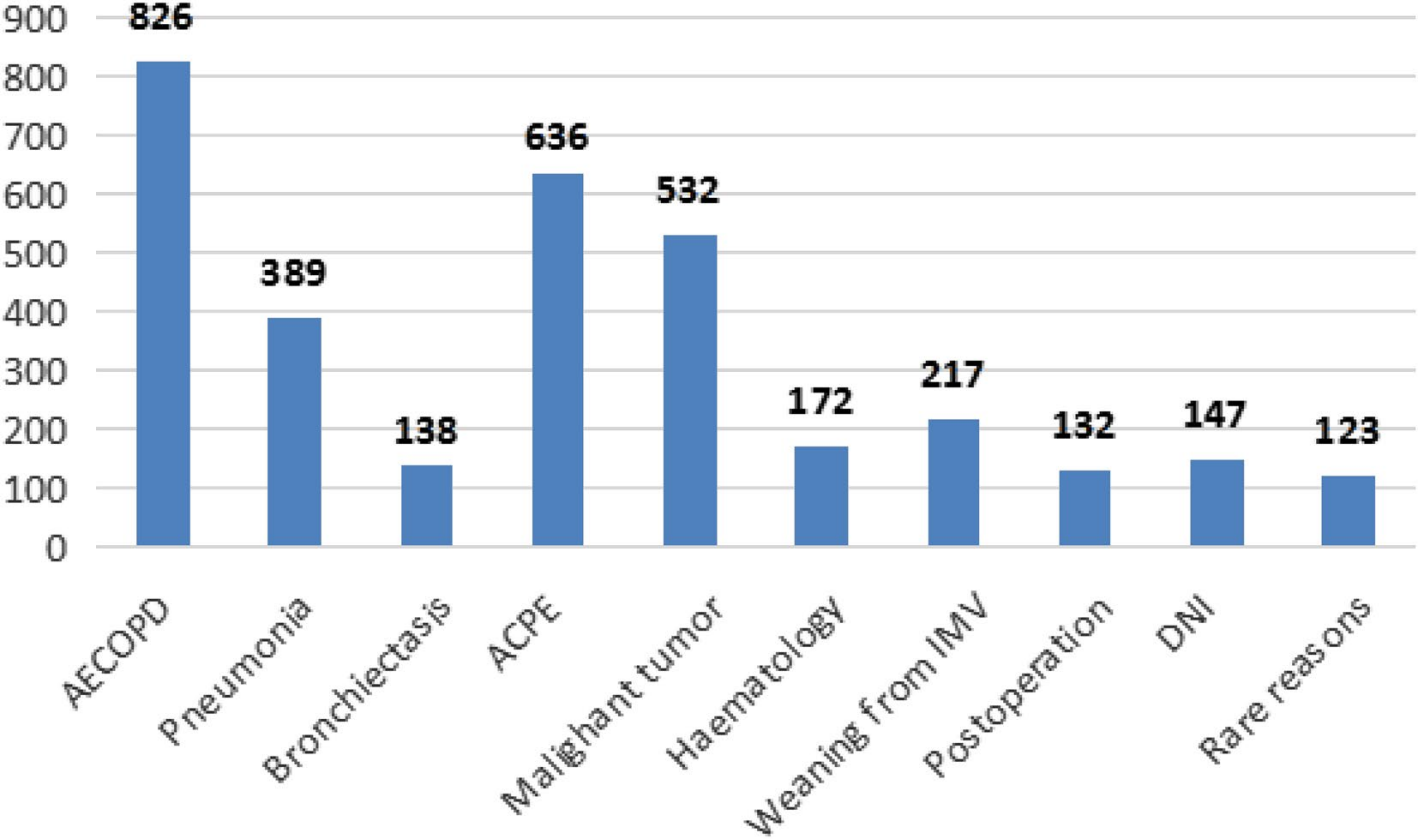
Reasons for NPPV treatment. *AECOPD* acute exacerbation of chronic obstructive pulmonary disease, *ACPE* acute cardiogenic pulmonary edema, *IMV* invasive mechanical ventilation, *DNI* Do-not-intubate

In our study, the reasons for patients received NPPV treatment were 826 cases of AECOPD, 389 cases of pneumonia, 138 cases of bronchiectasis, 636 cases of ACPE, 532 cases of malignant tumor complicated with pulmonary infection, 172 cases of haematology complicated with pulmonary infection, 217 cases of weaning from IMV, 132 cases of postoperation for heart, chest, abdomen and limb fractures, 147 cases of do-not-intubate (DNI) due to old age or critical illness, and 123 cases of rare reasons, such as neuromuscular diseases, asthma, OHS and chest wall diseases (Fig. 2).

### General clinical data of the success group and the failure group

Among 3312 patients, there were 2025 patients in the success group and 1287 patients in the failure group. No statistical differences were detected in terms of patients' age, gender, use of analgesia and/or sedation and complicated with barotrauma between the two groups (*P* > 0.05).

Patients in the success group showed significant higher serum albumin levels (34.93 ± 5.30 vs 32.52 ± 5.80 g/L, *P* < 0.01), higher Ca^2+^ concentration (2.13 [2.03, 2.24] vs 2.09 [1.96, 2.21] mmol/L, *P* < 0.01), lower blood glucose levels (6.24 [4.99, 8.32] vs 6.56 [5.12, 8.79] mmol/L, *P* < 0.01) and longer LOS (16 [11, 25] vs 14 [8, 23] days, *P* < 0.01) compared with the failure group.

### Characteristics of NPPV application

There were no statistical differences in IPAP and EPAP (14 [12, 15] vs 14 [12, 15] cmH_2_O, 5 [4, 6] vs 5 [4, 6] cmH_2_O; *P* > 0.05) between the success and failure groups. Duration of NPPV treatment in the success group was significant longer than that in the failure group (5 [2, 10] vs 2 [1, 5] days, *P* < 0.01) (Table 1).

**Table 1:** Comparison of clinical data between the success and failure groups

	NPPV-success (*n* = 2025)	NPPV-failure (*n* = 1287)	*t/χ*^2^/*Z*	*P* value
Age (years)	67.82 ± 15.01	68.24 ± 15.48	− 0.754	0.451
Gender (n): male	1368	882	0.316	0.574
female	657	405		
Analgesia and/or sedation (n): yes	43	26	0.039	0.843
no	1982	1261		
Barotrauma (n): yes	10	9	0.587	0.444
no	2015	1278		
IPAP (cmH_2_O)	14 (12, 15)	14 (12, 15)	− 0.101	0.920
EPAP (cmH_2_O)	5 (4, 6)	5 (4, 6)	0.355	0.723
Albumin (g/L)	34.93 ± 5.30	32.52 ± 5.80	12.03	< 0.001
Ca^2+^ (mmol/L)	2.13 (2.03, 2.24)	2.09 (1.96, 2.21)	− 7.148	< 0.001
Blood glucose (mmol/L)	6.24 (4.99, 8.32)	6.56 (5.12, 8.79)	2.969	0.003
Duration of NPPV treatment (days)	5 (2, 10)	2 (1, 5)	− 17.267	< 0.001
Length of hospital stay (days)	16 (11, 25)	14 (8, 23)	− 6.566	< 0.001

### Multivariate logistic regression analysis of the efficacy of NPPV treatment

Univariate analysis showed that there were significant differences in serum albumin levels, Ca^2+^ concentration, blood glucose levels, duration of NPPV treatment, and LOS between the success and failure groups (*P* < 0.05). Subsequently, multivariate logistic regression analysis was conducted on the above data to select independent risk factors for the efficacy of NPPV treatment. The results demonstrated that serum albumin levels and duration of NPPV treatment had statistical significance for the efficacy of NPPV treatment (*P* < 0.05) (Table 2).

**Table 2:** Multivariate logistic regression analysis of the efficacy of NPPV treatment

variable	*β*	Standard error	Wald	*P* value	OR (95%CI)
Age (years)	− 0.001	0.003	0.112	0.738	0.999 (0.994–1.004)
Gender (*n*)	0.067	0.080	0.699	0.403	1.070 (0.914–1.252)
Analgesia and/or sedation (*n*)	− 0.248	0.265	0.876	0.349	0.780 (0.464–1.312)
Barotrauma (*n*)	0.629	0.504	1.559	0.212	1.876 (0.699–5.039)
IPAP (cmH_2_O)	− 0.010	0.017	0.386	0.534	0.990 (0.958–1.023)
EPAP (cmH_2_O)	0.018	0.032	0.331	0.565	1.018 (0.957–1.083)
Albumin (g/L)	0.075	0.007	117.228	0.000	1.078 (1.063–1.093)
Ca^2+^ (mmol/L)	0.031	0.043	0.523	0.469	1.032 (0.948–1.123)
Blood glucose (mmol/L)	− 0.003	0.004	0.377	0.539	0.997 (0.989–1.006)
Duration of NPPV treatment (days)	0.086	0.008	127.385	0.000	1.089 (1.073–1.106)
Length of hospital stay (days)	0.002	0.003	0.367	0.545	1.002 (0.996–1.009)

## Discussion

In our study, the failure rate of NPPV treatment was 38.86%, which was in keeping with that reported by the previous literature (5%–40%) [15]. Univariate analysis showed that patients' age, gender, use of analgesia and/or sedation, complicated with barotrauma, IPAP and EPAP were not the influencing factors of the efficacy of NPPV treatment. However, serum albumin levels, Ca^2+^ concentration, blood glucose levels, duration of NPPV treatment and LOS were the influencing factors of the efficacy of NPPV treatment. Multivariate logistic regression analysis indicated that serum albumin levels and duration of NPPV treatment were independent risk factors for the efficacy of NPPV treatment.

Serum albumin is an important parameter in clinical assessment of nutritional status of patients, and hypoalbuminemia is considered as a marker of malnutrition-inflammatory response syndrome [16]. The reduction of albumin levels can lead to a series of adverse effect, such as reducing anti-infection ability, weakening respiratory muscles, decreasing ventilation function, declining lung function. Thus, if the patients' albumin levels are low, it is likely to affect the efficacy of NPPV treatment and prolong or even worse the weaning [17]. It had been reported that hypoalbuminemia was one of the strongest indicators of poorer prognosis in patients with long-term mechanical ventilation [7]. Pacilliet et al. [5] found a significant value for pneumonia and serum albumin levels in patients with NPPV treatment failure, and serum albumin levels in the failure group were significantly lower. The results of our study confirmed that serum albumin levels of the success group were higher than that of the failure group, suggesting that low serum albumin levels were the risk factor for NPPV treatment failure, and multivariate logistic regression analysis indicated that low serum albumin levels were the independent risk factor, which was consistent with the above studies. The main causes of hypoalbuminemia in patients are insufficient intake and increased body consumption. The previous study declared that reasonable protein intake was closely related to the improvement of clinical outcomes in critically ill patients, and 1.2–2.0 g·kg^−1^ (actual body weight) ·d^−1^ was recommended to estimate the protein requirement of critically ill patients [18]. Comher et al. [19] also suggested that protein intake ≥ 1.2 g·kg^−1^ (actual body weight) ·d^−1^ could reduce the mortality rate and shorten LOS of ICU patients. Therefore, reasonable and effective nutritional support is extremely important, which can provide the body with energy supply, improve the immunity of patients, enhance respiratory muscle strength, restore lung function, and increase the success rate of patients treated with NPPV.

Serum calcium has several essential physiological roles including cell signal, neurotransmission, muscle contraction, as a cofactor in enzymatic reactions, and can regulate the inflammatory response in critically ill patients [20]. Zhang et al. [21] studied 15,409 ICU patients and found that Ca^2+^ concentration in the non-survival group was significantly lower than that in the survival group (*P* < 0.001). Ca^2+^ concentration in normal on admission was associated with lower mortality, and mild hypercalcemia (1.25–1.35 mmol/L) or moderate hypocalcemia (0.8–0.9 mmol/L) was associated with increased risk of death. In this study, Ca^2+^ concentration in the success group was higher than that in the failure group, suggesting that patients with low Ca^2+^ concentration had a higher failure rate of NPPV treatment. Therefore, clinicians need to reasonably adjust Ca^2+^ concentration in critically ill patients to improve the clinical outcome.

In this study, there were 724 patients with diabetes, while 1801 patients with hyperglycemia on admission were far higher than the number of diabetic patients. Chakrabarti et al. [10] believed that hyperglycemia, even when found at only one time point, related to the success or failure of NPPV treatment regardless of previous diagnosis of diabetes or use of glucose-influencing drugs such as insulin or glucocorticoids, and the initial hyperglycemia had independent prognostic value. The results of our study showed that blood glucose levels of the success group were lower than that of the failure group, and the median of blood glucose levels in the two groups was above the normal level. In addition, Dimoulis et al. [22] found that insulin sensitivity and glucose metabolism were improved along with the improvements of respiratory failure after NPPV treatment. Based on the analysis above, clinicians should improve patients' cognition of the harm of hyperglycemia, strengthen the management of hyperglycemia, and control blood glucose levels within the normal range quickly and safely, so as to minimize the impact of hyperglycemia on the therapeutic effect of NPPV.

Steriade et al. [8] revealed that duration of NPPV treatment and LOS were related to the efficacy of NPPV treatment (*r* = 0.372, *r* = 0.432; *P* < 0.001). The results of present study showed that duration of NPPV treatment and LOS in the success group were longer than those in the failure group, and multivariate logistic regression analysis showed that duration of NPPV treatment was the independent risk factor for the efficacy of NPPV treatment. Contou et al. [23] reported that the patients with PaCO_2_ > 60 mmHg, compared with the patients with PaCO_2_ ≤ 60 mmHg, required longer duration of NPPV treatment and LOS. However, Chen et al. [24] reported 188 patients who received prophylactic NPPV after extubation, according to duration of NPPV treatment, were divided into prolonged (duration of NPPV treatment > 3 days) and non-prolonged groups (duration of NPPV treatment ≤ 3 days). The results made clear that compared with non-prolonged group, prolonged group significantly increased the risk of complications, prolonged ICU and hospital stay, but had no significant effect on mortality. Correa et al. [9] identified that ICU and hospital stay of NPPV failure patients were significantly longer than those of NPPV success patients (*P* < 0.05). It is inconsistent with the results of our study, which may be due to the different underlying diseases of patients, more severe hypercapnia, and the application of analgesia and/or sedation in a small number of patients. Duan et al. [25] believed that a DNI order and pH ≥ 7.35 were independent risk factors for prolonged NPPV treatment at the beginning of NPPV, while tachycardia and low oxygenation were other independent risk factors for prolonged NPPV treatment at days 1 and 7 in patients who were still on NPPV. Nevertheless, the above research may still not be able to explain why duration of NPPV treatment and LOS in the success group were longer than those in the failure group. We hope that more clinical studies can provide a clear answer in the future.

This study has some limitations to be acknowledged. First and foremost, it is a single-center study with limited target population, and the results may be different from those of other centers. Moreover, this study was a retrospective study, and the observation indexes for the therapeutic efficacy of NPPV were not comprehensive enough. Furthermore, the lack of APACHE II score, GCS score, complications (i.e., ventilator-associated pneumonia, skin injury, patient-ventilator asynchrony) and so on may lead to the bias in the results of the study.

## Conclusion

NPPV has been widely used and its utilization rate is increasing year by year. This study suggests that patients' age, gender, use of analgesia and/or sedation, complicated with barotrauma, IPAP and EPAP are not correlated with the efficacy of NPPV treatment. Serum albumin levels, Ca^2+^ concentration, blood glucose levels, duration of NPPV treatment and LOS are correlated with the efficacy of NPPV treatment, among which serum albumin levels and duration of NPPV treatment are independent risk factors for the efficacy of NPPV treatment in respiratory failure.

## Data Availability

The data set supporting the results of this article are included within the article.
